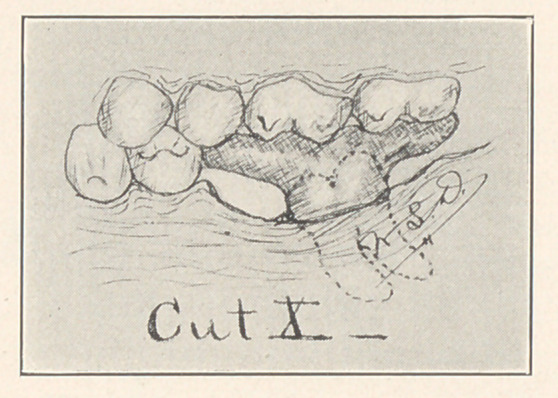# Dental Bridge and Pier Construction

**Published:** 1903-03

**Authors:** William Slocum Davenport

**Affiliations:** Paris


### International Dental Journal.

Vol. XXIV. March, 1903. No. 3.

### Original Communications.[1]

### DENTAL BRIDGE AND PIER CONSTRUCTION.[2]

BY WILLIAM SLOCUM DAVENPORT, D.D.S., PARIS.

In the science of dental articulation we have established laws which govern the movements and relations of the teeth to the dental arch. The principles of interlocking, counterbalancing, and self-retention of the teeth hold good in the dental arches, even though restored by bridges or other artificial means.

I endeavored to show at the International Dental Congress, also at the Cologne meeting of the American Dental Society of Europe, that a successful bridge depended far more upon proper articulation than on its immediate anchorage. (See *Items of Interest,* July and October, 1901, also report of International Dental Congress, 1900.)

Nearly all failures in bridges can be attributed either to the faulty articulation which allows the bridge to become displaced

---

[1] The editor and publishers are not responsible for the views of authors of papers published in this department, nor for any claim to novelty, or otherwise, that may be made by them. No papers will be received for this department that have appeared in any other journal published in the country.

[2] Read before The New York Institute of Stomatology, November 6, 1902.

*en masse* or to the fact that one or more of the anchors loosen, exposing the pier teeth to decay. A bridge properly articulated often continues to perform its functions long after its anchors have loosened and the piers decayed away. Experience and close observation have convinced me that a greater proportion of the bridges would be better anchored to one tooth only, with a compensating articulation, or a pier on which one end of the bridge rests loosely, thus permitting the normal movements of the teeth and allowing for all forces.

"A bridge is not stronger than its weakest part." Engineering principles must be so applied that the weaker piers will not undergo destruction through unnatural strain and faulty protection. With this idea in view, I present for your consideration over thirty bridge cases from practice which were anchored firmly to but one pier.

I am not trying to condemn multiple anchorages or "classic methods," but only wish to show some of the faults which have justly prevented bridge-work from occupying the place it should in dentistry.

In the making of caps and bands for bridges I think I have secured, by the use of gold, platinum, and iridium, accurate adjustment, maximum strength, and minimum size. With these three qualities we are able to do away almost entirely with the grinding of the natural teeth, especially in the region of the articulation. By the perfect adaptation and great stability of this cap it can be used as a complete cap or an open cap, whichever may best accommodate itself to the articulation of the teeth and to æsthetic conditions. For convenience I give the following names of the parts used in bridge-anchors, the letters indicate the various applications in the illustrations.

A. Rigid cap.
B. Part rigid and part pliable cap.
C. Contour wire.
D. Anchor pivot.
E. Retaining anchor pivot.
F. Ball pier.
G. Ball-lock pier.
H. Socket pier.
M. Concave pier.
N. Bridge lug.

Cut_I_

Cut_II_

RIGID CAP.

In the construction of the rigid cap a plaster model is made from an impression of the tooth taken with modelling compound. A strip of pure platinum plate, thirty-three gauge (Cut I, 1), is fitted to the convexity of the plaster tooth and soldered with pure gold (see Cut I, 2). The edge of this band is slit down in the old way, and burnished and pressed to the tooth by means of a cork. Encircling the platinum cap a platinum contour wire, twenty-six gauge, is fitted in the desired place and all soldered lightly with pure gold (see Cut I, 3). The band with wire attached is verified on the natural tooth; the defects in fitting are corrected by burnishing the soft thin metal perfectly to the tooth. The wire at this stage is useful as a point of contact in pushing the band to its place and in removing the same.

Platinum and iridium gold clasp metal (Williams's preferred) is uniformly melted over the surface of the platinum band and about the wire, which later acts as a guide for the even distribution of the metal over the surface, making a very rigid band with beautiful contour, and at the same time very thin at its edges. (Cut I, 4). With a little experience investment of the band for soldering is not necessary, owing to the fact that the natural tendency of the melted metal is to form itself about the wire, thus preventing its going inside the band and destroying the fit, unless an excessive quantity of the metal is used. When desired, a quick investment for this work is to pack the cap as full as possible with dry asbestos powder and solder at once. For the history and further study of the construction of this cap, see paper by the author published in the INTERNATIONAL DENTAL JOURNAL in 1894 (Vol. XV., page 441).

PART RIGID AND PART PLIABLE CAP.

The part rigid and part pliable cap (Cut I, 5) can be accurately adjusted to any tooth without previously destroying its natural contour. This cap is made in the same manner as the rigid cap (Cut I, 4), excepting that it is first fitted to the plaster tooth from bulbous portion to masticating surface. The platinum guide-wire is made to encircle the platinum at the greatest convexity of the tooth. The band is strengthened by melting small pieces of rigid metal between the wire and masticating surface,—the wire acts as a boundary-line in creating the part rigid and part pliable cap. The more rigid or stronger part of the band is about the point between the tooth's convexity and the masticating surface (Cut I, 5, J), while the pliable part of the band (Cut I, 5, K) permits its being passed over the convexity of the tooth and afterwards burnished about the neck of same or into any groove or hole that may have been made as a means of retention.

### CONTOUR WIRE FOR DENTAL CONTACT.

Cut II illustrates another use for the contour wire. When a contact is required with the neighboring teeth in bridge-anchorages or regular crowns, proceed as in Cut I, 3, excepting where dental contact is required leave the wire free to be adjusted to the tooth, as in Cut II, C, and tack it with pure gold at the sides.

The wire in that position acts as a guide in distributing the melted metal in desired quantity and place, making the dental contact. I might add that the unpolished work for your inspection is as the blow-pipe left it, which shows better the details of construction and the forms obtained by the melted metal.

### ANCHOR PIVOT.

Cut III, D, shows the construction of an anchor pivot. To conform to the articulation of the teeth it is usually necessary to concave the tooth by grinding at the lingual surface. In the concavity burnish a piece of platinum or gold plate, through this pass a pivot, remove the two, and solder; replace, and take an impression in the same manner as for an ordinary pivot tooth; wax the pivot to the impression, pour the model with soldering plaster, and wax about the cap when the teeth are being placed; build this up to the desired thickness with solder during the process of soldering the bridge. A similar method of using an anchor pivot was published by Dr. J. Leon Williams (*Dental Cosmos,* 1900, page 738).

### RETAINING ANCHOR PIVOT.

Cuts III and VII, E, illustrate a retaining anchor pivot. This pivot is made in the same manner as the anchor pivot, excepting that it has an artificial cusp which serves, by its manner of articulation with the opposite teeth, to prevent the tooth to

D
E
Cut III

F
A
Cut IV

Cut-V-

Cut-VI-

which it is attached from being driven outward. This cusp is made in the same manner as the articulating surface of the adjoining teeth, details of which will follow.

### BALL PIER.

Cut IV, F, shows a diagram of a ball pier, also the manner in which it is applied. A pivot extends up the root with a cap protecting the end. A bridge with a rigid cap is cemented to the molar at the back and extends forward around the neighboring molar by means of a strong iridium platinum bar. The bridge is made with a socket so arranged that it fits loosely over the ball pier, thus forming a ball-and-socket joint which performs all its functions with comparatively little strain to the root. I am indebted to Dr. Hurlbut, of Cannes, for the idea of extending the bridge around a molar.

### BALL-LOCK PIER.

Cut V shows the details of the ball-lock pier. The construction is nearly the same as the ball pier, excepting that a key or a screw is made to pass through a hole in the outer cap (or bridge part) and through the groove in the *ball* of the inner cap, thus locking the two caps together.

When movement is required about the ball-lock pier, as in the case where it is used as the second pier to a bridge, the groove in the ball must be made large enough to permit a slight movement; but should the ball-lock pier be used as first ball of a bridge or as the anchor of a crown, the key should be fitted tightly in the ball groove, thus locking the two caps tightly together.

### SOCKET PIER.

Cut VI, H, illustrates another form of utilizing a frail root as a pier for one end of a bridge. A gold cap with a socket pivot is cemented to the root. The bridge is cemented to the molar only by means of a short rigid cap which extends to the bulbous portion of the tooth. The other end of the bridge terminates with a ball-like end, which rests loosely into the socket cap pier, forming a ball-and-socket joint.

### CONCAVE PIER.

Cut VII, M, is a good illustration of a concave pier. In this case it is a simple concavity made in the retaining anchor pivot.

The convexity in the natural teeth or one ground in the filling answers the same purpose as a rest for the free end of the bridge.

BRIDGE LUG.

Cut VII, N, shows a bridge lug which is a very important feature of single anchored bridges. They are made by fitting a small piece of pure gold plate into the concave pier. To prevent the piece of gold being displaced in soldering, a bit of wire or flat gold is soldered to it in such a way that it is held in the desired place by the investment. The bridges which I present (Cuts VII, VIII, IX, and X) illustrate some of the principles of bridge-anchorage that I wish to bring forward.

Fig. 1 shows the left posterior single extension bridge anchored by means of a rigid cap or ring on molar at one end, with a lug at the other end resting in a concave filling of the bicuspid.

Fig. 2. Left anterior double extension bridge. Anchored in the middle with an anchor pivot entering the canal of canine; back extension rests in the natural concavity in bicuspid.

Fig. 3. Right anterior single extension bridge anchored by means of retaining anchor pivot extending into canal of canine.

Fig. 4. Right posterior double extension bridge anchored to first molar by means of part rigid and part pliable cap, with a lug of the posterior extension resting in concave filling in wisdom-tooth; anterior extension rests in concave pier which was previously made in retaining anchor pivot of Fig. 3.

BRIDGE IMPRESSIONS.

An impression of the teeth with the anchors in place is taken with modelling compound slightly softened. To prevent drawing, the compound should not be too tightly forced in about the necks of the teeth and should be made hard in the mouth by means of cold water. Sectional impressions are advisable when accuracy of the gum is required; the anchors are removed from the teeth and fastened into the impression by first cutting a little of the compound away at the side of the anchor and filling in with melted stick wax. The impressions are poured with soldering plaster.

BRIDGE ARTICULATIONS.

The Bonwill articulator is indispensable in bridge-work. It shows minutely where protection must be given to facings, also

Cut. VII

Cut. VIII

Cut IX

Cut X

how the planes of articulation must be made to give the greatest amount of masticating and retaining functions. The porcelain facings are ground and backed to their points and placed in the desired position on the model, the shape of the articulation surface is made in wax, and afterwards reproduced by burnishing pure gold plate, thirty-three gauge, directly to the surface of the wax.

### SETTING BRIDGES.

In retaining bridges the greatest aid is to have sufficient strength and perfect adaptation of the anchor. When this is secured, it matters but little what are the materials used in setting a bridge. I usually use Gilbert's gutta-percha or Eisfelder's No. 7 cement, as the case suggests; although I have had (under favorable conditions) successes from the use of chloropercha, combination of chloropercha and cement, and combination of cement and amalgam.

I have shown in detail my method of making strong and accurately fitting bridge-anchors and also the preservation of piers in general.

My critics have doubted the possible success of single anchor bridges on the ground of lateral pressure and excessive leverage. For the benefit of those, I would refer them to the well-known works on the science of dental articulation.

As I have before stated, the principles that hold good for a single tooth in the arch are the same for a bridge. It becomes only a question of proportion.

Cut X illustrates a double extension bridge anchored to a molar by means of a part rigid and part pliable cap.

The bridge forms a contact with the first bicuspid and articulates with one bicuspid and two molars.

As for the success of this class of bridges, I have a good number which have performed their functions from months up to a few years, and, when made properly, have not given the slightest discomfort or inconvenience.

For further consideration of this subject, I call your attention to the models on exhibition taken from over thirty cases from practice where the bridges were anchored firmly to but one pier.

## Figures and Tables

**Cut. I. f1:**
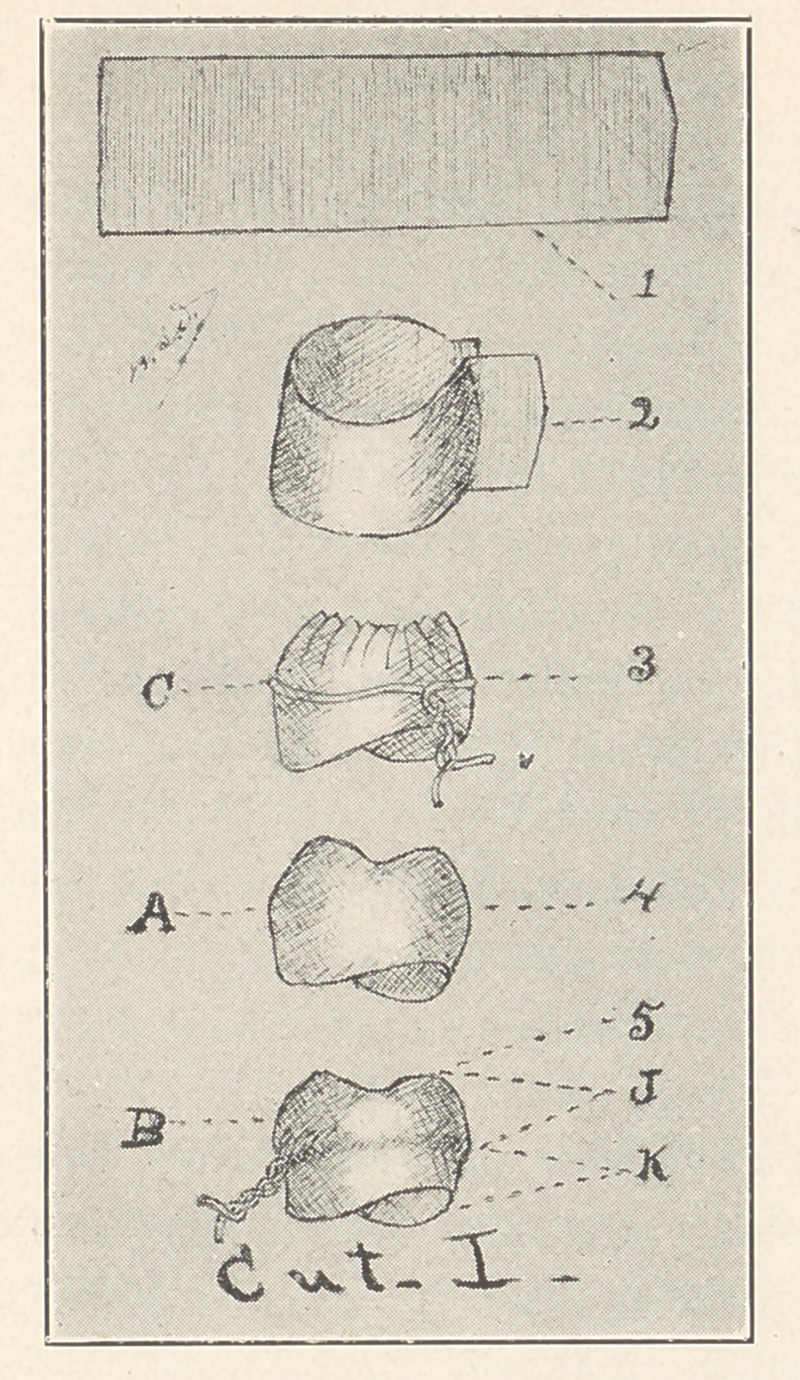


**Cut. II. f2:**
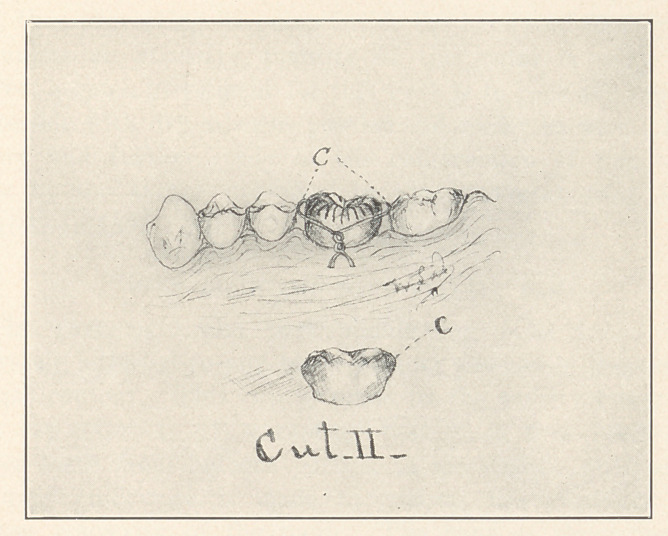


**Cut. III. f3:**
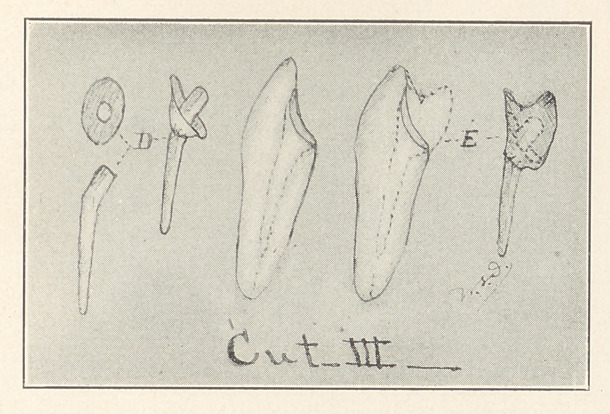


**Cut. IV. f4:**
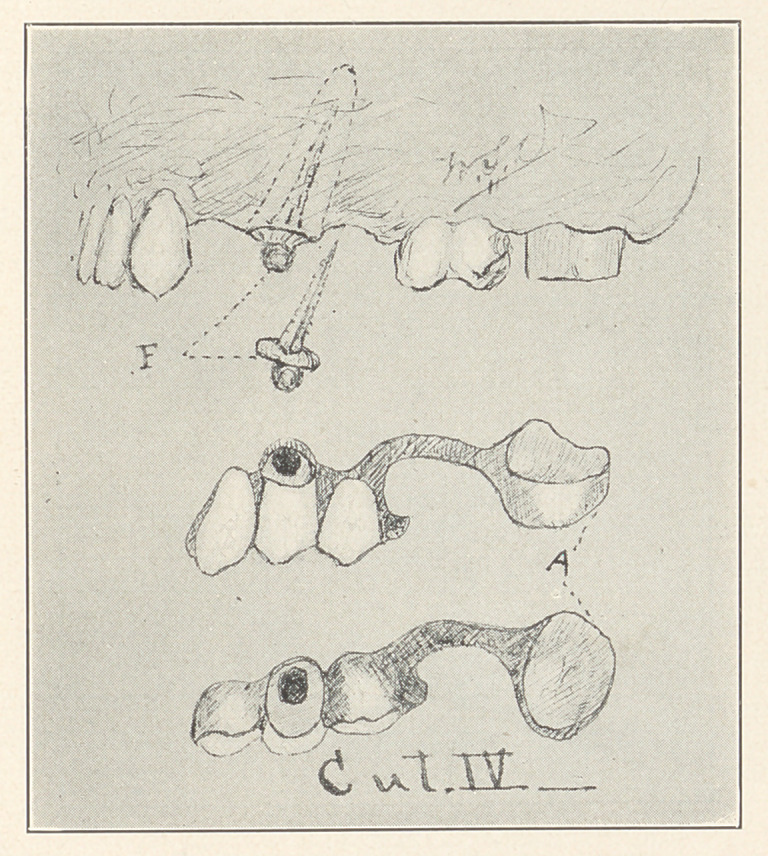


**Cut. V. f5:**
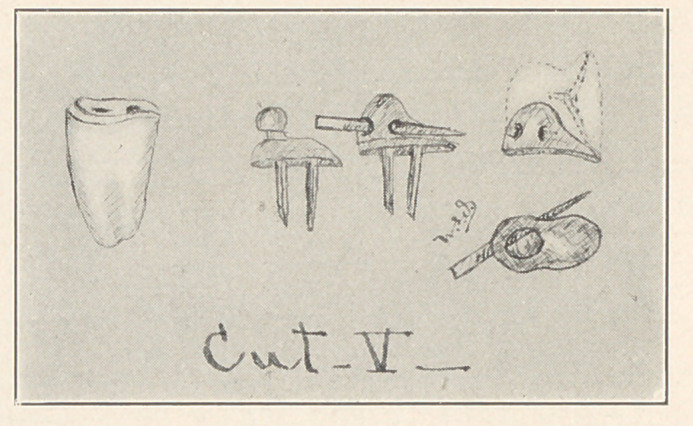


**Cut. VI. f6:**
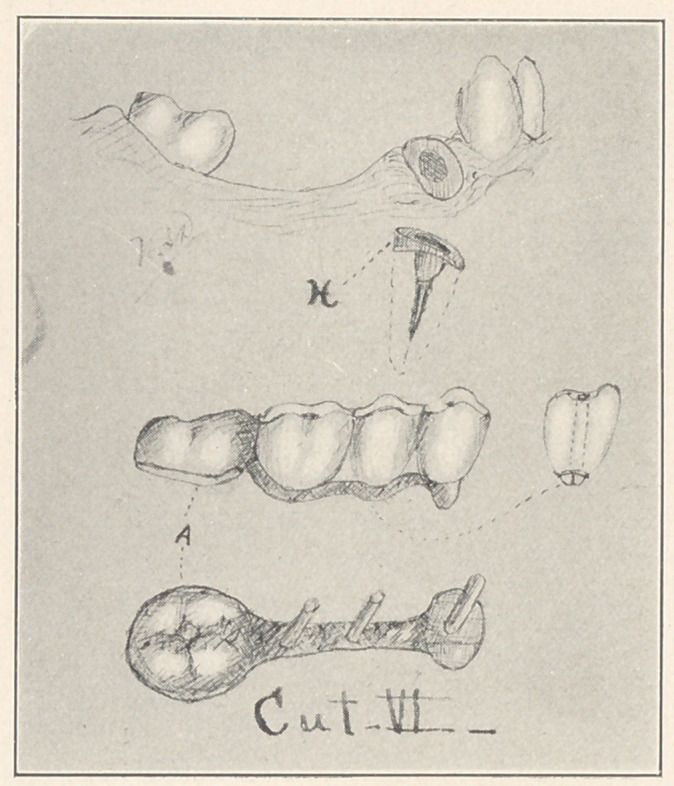


**Cut. VII. f7:**
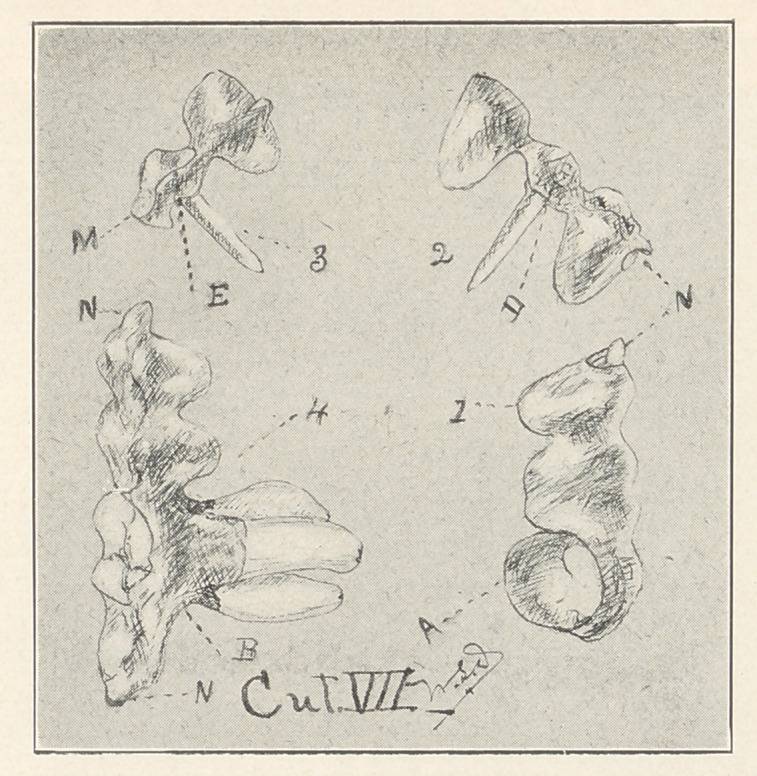


**Cut. VIII. f8:**
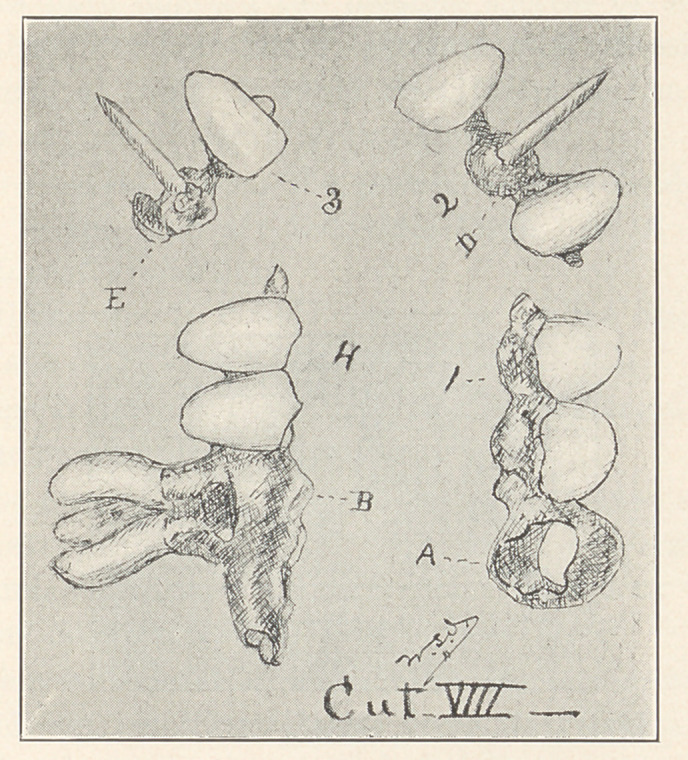


**Cut. IX. f9:**
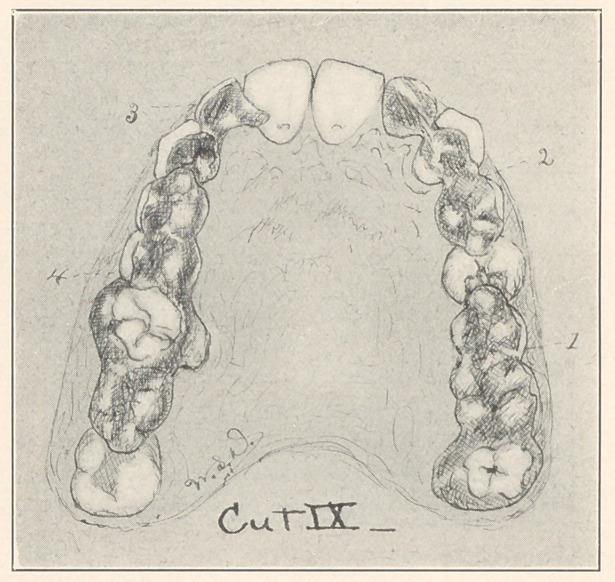


**Cut. X. f10:**